# Understanding perceived access barriers to contraception through an African feminist lens: a qualitative study in Uganda

**DOI:** 10.1186/s12889-021-10315-9

**Published:** 2021-02-02

**Authors:** Meghan A. Potasse, Sanni Yaya

**Affiliations:** 1 School of International Development and Global Studies, Faculty of Social Sciences, University of Ottawa, Ottawa, Canada; 2 The George Institute for Global Health, Imperial College London, London, UK

**Keywords:** Contraception, Family planning services, Healthcare personnel, Health services accessibility, Focus groups, Uganda

## Abstract

**Background:**

There are many barriers that impact a woman’s access to contraception in rural sub-Saharan Africa, such as financial constraints, supply shortages, stigma, and misconceptions. Through and African Feminist lens, this study examines how these perceived barriers intersect with each other, and how they negatively impact women’s access to family planning and their perceived value of contraceptives in Luweero, Uganda.

**Methods:**

This qualitative study analyzed data collected from healthcare workers at one private clinic and one public clinic that offer family planning services in four focus group discussions in Luweero, Central Region, Uganda. Two focus group discussions were held in each clinic. Eligible participants spoke English, were at least 18 years of age, and had at least 3 years of experience as a healthcare worker in Luweero. Among the participants were nurses, midwives, family planning counsellors, and village health workers, both male and female. Coded transcripts were analyzed using a reflexive methodology through an African Feminist lens.

**Results:**

Most of the responses indicated that financial constraints experienced either by the clinic or the women significantly impact access to family planning. Certain social barriers were discussed, and the participants explained that barriers such as stigma, misconceptions, lack of knowledge, religiosity and cultural values impact women’s motivation or ability to access contraceptive methods. Side effects also have a significant role to play in women’s ability or motivation to navigate through these perceived social barriers.

**Conclusions:**

Participants determined that increased funding for transportation for village health teams, consistent funding for free contraception, and expanded sensitization efforts that particularly target men would be some of the most impactful methods they can adapt to address some of these barriers.

**Supplementary Information:**

The online version contains supplementary material available at 10.1186/s12889-021-10315-9.

## Background

The United Nations Sustainable Development Goals are global development targets aimed at improving the quality of life for all. Among these goals is the objective to achieve gender equality for women and girls, which includes the right to universal access to reproductive and sexual healthcare, such as modern contraception [1]. While the authors recognize that people of many different genders have childbearing capacity, the term ‘woman’ is used throughout this text due to the socio-cultural nature of this study. Recognizing that many communities in Africa still use traditional methods of contraception such as the pullback method or herbs and teas, for the purpose of this study modern contraception refers to hormonal birth control such as the pill, vaginal ring, injection, implant, IUD and patch. The question about the accessibility of modern contraception has been a topic of serious debate in the international development community, especially in the Sub Saharan African context where fertility remains very high [2–4]. Uganda’s exceptionally high birth rate is one of the highest in the world at 5.8 children per woman of child bearing age [5]. Research has demonstrated that there is an unmet need for contraception among women of childbearing age in rural Uganda [6–9]. Approximately 41% of women of childbearing age face barriers to seeking contraception, particularly for women living in rural areas [8]. For the most part, barriers result from a lack of community engagement and education, financial constraints and shortages of supply [7, 10]. Various barriers that affect women on financial, social and personal levels cause this unmet need, as well as an overall shortage of funding and resources within Uganda’s healthcare system [7, 10].

While there are governmental policies in place that give Ugandan women the right to access contraception, poor implementation of these policies is keeping reproductive and maternal health indicators low [4]. The rates of modern contraception use among youth are especially troubling, as 63% of unmarried sexually active women between 15 and 19 years old, and 43% of unmarried sexually active women between the ages of 20 and 24 years old are not using any type of birth control at all [8]. The overall rate of unmet need for contraception, that is women who want to either stop or delay pregnancies and cannot get access, is 31% in Uganda, despite the average in East Africa being 24% [11]. A reduction in unmet need of contraception would reduce the occurrence of unwanted pregnancies, meet the fertility desires of women in rural areas, improve overall community health, and serve national population policy goals [4, 7, 11–13].

Understanding why this unmet need exists requires a culturally sensitive analysis of the various perceived barriers that impact women economically, psychologically, and physically, preventing them from accessing contraception. Research has shown there are many sociocultural factors that can inhibit a Ugandan woman’s access to contraceptives [10, 14, 15]. A women’s motivation or ability to use family planning may be impacted if she has not born any or few boys or due to religious prohibition. Marital obligations also have an impact on a woman’s ability to access contraception. For example, it is highly desirable for men to have a very fertile wife, and bearing many children is a sign of respect towards the husband [10]. Gender inequality has a significant impact on the prevalence of modern contraception use among rural women. Consequently, the power imbalances that result from gender inequality often inhibits women’s ability to negotiate safe sex, therefore putting her more at risk for unintended pregnancy or sexually transmitted infections such as HIV/AIDS [16].

In addition to socio-cultural factors that can inhibit contraception accessibility, there are also numerous misconceptions about modern methods of birth control, and some women choose to use traditional practices as methods of contraception such as herbs [14]. One such myth is that modern contraception can cause permanent infertility, which is understandably a significant fear for many families that value fertility very highly, and for women who fear they may be unable to bear children and their husbands will leave them. Other misconceptions about side effects are the birth of abnormal babies, delay of returned fertility and even tumours and cancer [13]. In addressing these misconceptions, education outreach programs are integral and can lead to increased access to contraception and other safe sex practices, especially for younger people [17].

Finally, it is clear that the financial burden of contraception is one of the main barriers that women and their families face when seeking family planning methods, with socio-economic differences between rural and urban women. Women of low socio-economic standing, particularly in rural areas, are less likely to use modern contraception, and have about twice as many children than wealthier families in urban areas [18, 19]. These findings show an issue of equity among different classes of women in their reproductive autonomy, and ability to fulfill their long-term fertility desires. Intersections of poverty, education and socioeconomic status may further determine a woman’s ability to afford and practice family planning [20].

The purpose of this study is to use qualitative methods to comparatively explore and understand low uptake of family planning methods through an African Feminist lens by identifying perceived barriers to contraceptive use at a public and a private clinic. Specifically, focus group discussions were held with healthcare workers at these clinics who are experienced in providing reproductive health services, including dispensing contraceptives and counselling women on family planning. Community healthcare workers and Village Health Teams (VHTs) are an essential aspect of healthcare in Sub-Saharan Africa, as they bridge the gap between healthcare services and rural communities who might otherwise struggle to access basic care [21, 22]. The participants had insight on the specific reproductive healthcare services in their district and offered valuable information about the needs specific to their respective clients. Findings from this research may ultimately contribute new insight on solutions to reduce access barriers and increase the frequency and consistency of modern contraception use in rural Uganda by identifying interventions that would address one or more of these barriers.

### Theoretical framework

As the research terrain is in Uganda on an issue that concerns reproductive justice, a feminist framework born from the African perspective is most appropriate. African scholars have developed such theories as African Feminism by Chikwenye Ogunyemi, Mary Kolawole’s Womanism, Malora Ogundipe-Leslie’s stiwanism (acronym of: Social Transformations Including Women in Africa), and Nnaemeka’s negofeminism- feminism of negotiation. These theories are all centered on the social and cultural constellations of African societies and are directed at changing the existing power relations between men and women [23]. It also questions features of “traditional African values without [unfairly criticizing] them, understanding that these might be viewed differently to the different classes of women” [24].

African Feminisms recognize men as partners in the struggle against gender oppression rather than as the enemy and emphasize the complimentary relationship between men and women rather than conflict. Gender roles are seen as asymmetrical, parallel, and autonomously linked in the circle of life [25]. On the other hand, while African feminism is not opposed to African culture or heritage, said culture cannot be immobilized in time to the advantage of men, as most men in African want it to be [26]. Conceptualizing gender within the parameters of African cultures allows African women to view their womanhood and African identity as integral and complimentary [27]. African feminisms center traditional religious and spiritual beliefs based on African oral histories and festivals which have women at the center of social order, custodians of earth, fire and water and men as guardians of their custodial rights [25]. The miracle of birth and motherhood, as well as the complimentary roles men and women play in reproduction and the continuity of humanity are recurring themes in African Feminist discourse. Finally, African Feminisms discuss issues surrounding gender in the context of other oppressive systems such as racism, neo-colonialism, imperialism, religious fundamentalism, socio-economic exclusion and exploitation as well as corrupt and dictatorial political systems, far exceeding the race-class-gender scheme of African American feminism [23, 28].

## Methods

### Research setting

Uganda was chosen for this study because it has one of the highest birthrates in the world, and research has demonstrated an unmet demand for contraception [29]. Luweero is in the central region approximately 60 km north of the capital, Kampala, with a population of over 476,000; most of the Ugandans in this district live in rural settings [30]. Two clinics were the focus of this study, the Reproductive Health Uganda (RHU) Luweero branch, and Shanti birth house. RHU is a publicly funded organization whereas Shanti is a private non-profit. Shanti and RHU provide various services to their communities, including education programs, maternal healthcare and delivery of family planning methods and education [31, 32].

### Research design and participant selection

This is a qualitative case study using focus group discussions as the method of data collection, and a focus group discussion guide as the main research tool [33]. Prior to the data collection, introductory meetings between the researcher and key stakeholders at Shanti and RHU were held. Study participants were recruited by word of mouth and the distribution of flyers to the head offices of the organizations and directly to the clinics. Participants were male and female healthcare workers with 3 years of experience in administering or prescribing contraception to women in Luweero and were of 18 years of age. Potential participants included doctors, nurses dispensing contraception, counsellors, and village health teams. Our findings were reported based on the Consolidated criteria for reporting qualitative research (COREQ).

### Data collection

Fieldwork was conducted by MP and took place between February and March 2020 at Shanti birth house and RHU in Luweero. There were four focus group discussions with a total of 27 participants. FDGs were stratified along gender lines in order to minimize the impact of gender inequality as dictated by cultural norms, which may cause some women participants to stifle their responses and listen to their male colleagues [34]. Participants were asked to attend one of the four focus group discussions that each lasted approximately 1 h. This number of discussion groups offers enough data to analyse all of the key themes, as Guest, Namey, McKenna found that 90% of all themes are discoverable in 3 to 8 FGDs [35]. A full description of focus group discussion guide is available (See Supplementary file 1).

### Data analysis

Qualitative content analysis was used in order to find themes in the dataset after the FDGs were redacted into a clean transcript edited for clarity [36]. Upon reviewing field notes as well as during the initial reviews of the recording, MP developed a coding scheme with which we would discover a pattern of themes within the dataset [37]. Nvivo 12 was used to aid in the data analysis stage, and MP incorporated the coding scheme into nodes, adding more and reorganizing them as the data was analysed.

Emerging themes were noted, and pertinent ideas and quotes were organized into meaning units [38]. These units were grouped into the coding scheme in Nvivo 12 where they were compared and contrasted and MP narrowed down the analysis in order to highlight the most significant themes in the dataset [37]. This is an inductive content analysis methodology and allows the analyse of a large set of qualitative data in order to discover the most significant access barriers for women seeking contraception in Luweero and how these issues can be addressed [39, 40].

### Trustworthiness

There must be evidence of methodological credibility of the findings in order for research to potentially have impact on policy and practice [41]. A reflexive methodology and theoretical framework was chosen as the primary researcher, MP, was socialized differently from the participants of the study, and this affects the author’s analysis and interpretation of the data [42, 43]. As the data set and passages were coded, the primary researcher also took handwritten notes in a project notebook on any key ideas, connections, questions or observations had as MP analysed the text [44]. This notebook was used throughout the planning stages of this project and as a reflective journal, developing an interpretation of the data through initial perceptions and analysing them under an African Feminist lens, consistently considering where personal biases were and where they come from. When MP was unsure how to code a passage, she analysed what meanings she saw in it and why she interpreted them that way. Methodologically, the journal was MP’s tool to continuously use reflexivity throughout the process, identifying where her social position is with relation to the research context, and reflecting on how that impacts her interpretation of the data [43].

### Ethical considerations

The ethical clearance approvals required for this project were obtained from the University of Ottawa’s Office of Research Ethics and Integrity and the Ugandan National Council for Science and Technology via the TASO Research Ethics Committee (REC) reference TASOREC/093/19-UG-REC-009. To ensure the confidentiality of the participants, personal identifiers were not included in the transcripts. Written informed consent for participation was obtained before the start of the audio recorded focus group discussions [45]. Participants were compensated 10,000 Ush for their time.

#### Findings

##### The impact of financial barriers on access to modern contraception

The cost of contraception impacts poor rural communities profoundly. Participants said the socio-economic well-being of the community in Luweero must be improved through measures that empower them economically, in order for more clients to be able to access healthcare in general. The contrast between the socio-economic contexts of urban and rural is explained in this quote from a participant:

“It [being unable to afford contraception] is very common in the rural areas. Because in the urban, the husbands are working and things in the urban are very expensive, but when you come this way, the little money, even if it [the price of contraception] has reduced, they are saying they cannot afford it. Because they are not working, or they cannot spend the 3,000 to get the contraception. But at least in the urban areas they know the cost of having those big families. And unlike in this community, they think that because they have a lot of food, they can provide enough children, so they don’t even want to lose the 3,000.” (Female participant, RHU)

Participant 1: “Maybe they lack money. Sometimes.”

Participant 2: “It can be up to 30 to 40 percent” (Female Participants, RHU)

The participants reported that 3000 USh is not as significant a cost for clients living in urban areas as there are more employment opportunities and it is far more difficult to manage in urban settings with large families. Consequently, women living in urban areas tend to have fewer children. In order to address these challenges, Shanti uses out-reach programs to extend access to isolated rural communities and provide information and short-term contraceptive methods for free. During these outreaches the healthcare workers also give their clients a contact at the clinic as well as their follow up appointment, which many are unable to meet due to transportation costs. While Shanti does not have a program whereby, they fund the transportation of their clients, they may fund the transportation of the VHTs in order for them to deliver short-term methods to their community. Additional costs to accessing contraception include pregnancy tests for clients who have not been on contraception for a prolonged period, STI screening and potential treatment for them and their partner, medications to manage side effects, and insertion and removal fees.

“Well at times when they come, and then like they can’t afford the method of their choice, mostly we tell them to wait for us in the community. There are times when we do the outreach, we give the methods at a free cost.” (Male Participant, Shanti).

“And there are expenses for example for the implant, when they are injecting the implant there is an extra charge for removing it.” (Female participant, Shanti)

### The impacts of side effects on the decision to use modern contraception

The side effects associated with modern contraception have significant psychological effects on the clients at both clinics participating in this study, impacting the operations of each clinic in similar ways. All four focus groups discussed these impacts and how as a result, side effects change clients’ trust and their perceptions of contraception’s value, despite receiving reliable information and being sensitized about the benefits. Some of the side effects mentioned were prolonged periods, cramping, pain from insertion or removal, lowered libido, and amenorrhea (absence of menstrual period). Fear of the side effects or the pain associated with insertion was reported as one of the most significant reasons as to why women are apprehensive about starting long-term family planning, or why they discontinue. This severely impacts the sensitization efforts of the clinics as well as their clients’ continuity of family planning, especially when clients have knowledge and information about the benefits of contraception yet discontinue because of the side effects.

“There are others who fear and don’t come because of the side effects. Because the other methods that can cause bleeding. And when someone goes into that, it feels like ‘ah it is inconveniencing’ and they get discouraged and they discourage others.” (Male participant, Shanti)

Participant 3: “To a certain level, some people will maintain that they don’t have the knowledge but the issue is about the side effects, which is the biggest of all of them because even those who have tried Jadelle, within a year or months they will come back and ask for it to be removed and when you ask why? Some hindrance bypass …”

Participant 2: “That is lack of knowledge”

Participant 3: “The knowledge is given, but side effects always can take away the knowledge.” (Male participants, RHU)

Other complications or illnesses such as fibroids or STIs are mistakenly associated with the side effects of modern methods, exacerbating the impacts of stigma and misinformation. Furthermore, as Depo-Provera is the most popular method, there are fewer misconceptions about the side effects of Depo than there are about other methods such as the IUD, which can cause women to perceive their options as limited, especially if Depo is out of stock. It is also convenient that Depo-Provera is a short-term method and clients feel more comfortable testing the effects of contraception on their bodies for a short period of time.

“Sometimes when people get these fibroids, even when they have not used family planning, but in case one has used family planning and gets it, they attach it to family planning.” (Female participant, Shanti)

Both RHU and Shanti have considered solutions to the issue of side effects as a motivational barrier for women considering family planning. Shanti recommends that medications to stop PV bleeding and counselling on side effects should be incorporated into their dispensing process. RHU explains that if a woman has side effects and needs medication to manage them, they can refer them to one of the healthcare centres where they may access them free of charge. Counselling on the side effects and misinformation about them during the outreach programs may also prevent women from discouraging each other in the community when they experience side effects. The clinics can also engage the VHTs to support women in the community struggling with side effects, as it would give clients easier access to the clinics’ services such as counselling and medication. Additionally, expanding the community’s knowledge about other methods may allow women to explore the risks and benefits of each type, thus providing them with more flexibility in their options so they may choose the method that would suit them the most.

“And maybe there are those getting side effects, so it can help when treating them (the side effects) are free. But if you charge money, they won’t continue. So, it means when they get a side effect like that, treatment is even free. That would encourage them to go on.” (Female participant, Shanti)

“Especially during … when a lady gets side effects, you first tell the VHT and share the issue.” (Male participant, RHU)

### Cultural values and religiosity in contrast to modern contraception

Cultural and religious values were reported to be a consistent reason why many women do not use modern contraceptives. As the participants explained, Luweero is situated in the Kingdom of Buganda where culturally, women are encouraged to give birth to literally all of the children in their womb, and large families are desirable. Having many children is also a way of honouring one’s husband and having many children is celebrated, particularly in rural areas. These cultural beliefs impact women’s motivation to begin family planning, as social norms may impact the value the community places on contraception, as high fertility is well respected and desired.

“This place belongs to a culture named Buganda whereby men have the tendencies to determine the number of children the wife should produce, so that is one hindrance.” (Male participant, RHU)

However, the participants reported that the standard of living of some of the larger families in the community are at risk because the parents are unable to provide enough for all of their children to receive basic education and be healthy. At times, parents are unable to provide basic necessities for their children, despite the desire to raise a large family.

“At least it would improve the lifestyle of the people and the community. Because people will be having the desired number of babies, they can take care of and raise, be able to pay fees, even improve the nutrition status at home. If you have a good amount of money which you can afford to buy more food you can even improve the eating habits, and the nutrition at home.” (Female participant, Shanti)

Participants reported that religious values from Catholic and Muslim communities also dictate that God has a plan for the number of children each woman has, and that using contraception is an affront against God’s will. For example, many also believe that every child is a blessing from God on the whole family. One of the participants from RHU describes:

“I think the Muslims think that even if you have 20 children, you can manage, or you can afford. But in reality, it is quite hard according to what we see on the ground. Because you find someone who has 20 children, that person says he can manage she can manage but according to what we see, they cannot manage.” (Female participant, RHU)

“Because there was a time we went for an outreach, somewhere in the Catholic area, and they were told that they considered our system to be obscene and misleading. Then you go to an area which is mostly occupied by Muslims, their religion does not attain that. You are supposed to have as many children as you can afford or as you can manage so they don’t allow it.” (Female participant, RHU)

This makes it particularly difficult for healthcare workers to navigate this context when they are attempting to sensitize the community about family planning. Shanti and RHU both reported their sensitization efforts are met with deep scepticism and often rejection in religious Catholic and Muslim communities, consistently impacting their operations and service delivery there.

### Misconceptions and stigma surrounding modern contraception

The participants from Shanti explained that rumours about the side effects or misconceptions of family planning circulate deep into villages, posing a significant challenge for healthcare workers. Some clients worry that the contraception will cause permanent infertility, abnormal babies, cancer or even death.

“Some say it will make you barren for life” (Female participant, Shanti)

“Others hear that they [implants] move, so they come to us and they are like ‘ah they told us when you use this method, then the capsule moves in the body and then it enters your heart and you die’, so we tell them no that is rumours it doesn’t move.” (Male participant, RHU)

Other ailments such as STIs, fibroids or other reproductive health complications may be erroneously attributed to modern methods of contraception, and some community members may spread rumours based on their personal experiences. RHU participants also explained that suspicion and distrust of the government also fuels misconceptions, as some community members may believe that the government has ulterior motives and wishes to cause infertility in young Ugandan women and girls. The participants noted that methods such as the IUD and implant suffer the worst reputation out of all the methods because they are more physically intrusive than many clients feel comfortable enough trying, unlike other methods like Depo-Provera, which is simply an injection that lasts 3 months. Depo-Provera offers a discreet, low risk, short-term method of reliable contraception; therefore, it is clear why it is the most popular method used at the clinics.

“They will use the method and go back saying they have just started a method, but it is making my private parts to itch. So, they combine their infections to the what? The method they chose.” (Female participant, Shanti)

Participant 5: “There are also rumours within the community that there is a hidden agenda for family planning methods” (Female participant, Shanti).

Participant 2: “Yes that maybe the government wants to produce some infertility to their young girls and women” (Female participant, Shanti).

“Some are also scared of the implant or IUD. Like the IUD sits in the uterus, and for that they are scared, and they are shy too. They are scared of the pain they think it is so painful.” (Female participant, Shanti)

Misconceptions about modern contraception contribute to the stigma surrounding family planning, which impacts the clients and the clinics’ outreach efforts in many ways. The social disapproval that stems from cultural norms and values, religiosity and misconceptions cause some clients to fear discrimination or shame unless they keep their contraceptives secret. Using family planning in secret adds risk for clients because being discovered could lead to social reprimand, exclusion or abuse. The stigma against contraception impacts the sensitization efforts of RHU and Shanti, as the participants explained:

“And people want these things done privately because their husbands, their neighbours, don’t want such things” (Female participant, Shanti)

“They don’t take it out of the house where the man can see them taking it. So, you find these are all kinds of risks” (Female participant, Shanti)

“Some of them forget to take the pill. Their husbands can come across it. There are other problems.” (Female participants, Shanti)

RHU and Shanti are often barred from speaking at schools about family planning because parents and teachers believe contraception will enable young girls to have their sexual debut at a young age or before marriage. Similarly, men may not wish their wives to practice family planning because they believe it may encourage their wives to be unfaithful and consequently, the outreach programs have low attendance rates, especially of men.

“There is opportunity to go and talk to the teen girls at the schools, but the parents don’t want to hear this. They say they have this fear that once these children are informed about family planning, they will go crazy and go start running after men. So those are our challenges we are facing.” (Male participant, RHU)

In the field, the VHTs have to navigate this stigma, and occasionally manage to discreetly deliver contraception to clients with limited mobility. At the clinics, the healthcare workers explained they become aware of certain misconceptions or stigmas when their clients ask them if something they heard in the community is true. It is critical for healthcare workers to seize this opportunity to counsel their clients sensitively on all of the facts surrounding risks, benefits, resources and choices available to them. Additionally, ensuring the dissemination of information in multiple forms of media such as radio and TV ads, illustrative pamphlets, newspaper articles and posters is widely available in the Lugandan language would make information more accessible the community.

“And they don’t come asking ‘is it … a,b,c,d?’ they come in and they tell you like ‘they told me this is this and this, is it true?’” (Male Participant, Shanti)

### Sensitizing men on modern contraception

The healthcare staff and VHTs explained that more women than men attend the sensitization outreach programs in the field. Sensitization programs specifically geared towards informing and educating men have not been running at RHU since 2018. As men are typically the head of the household in Uganda, they work and control the family finances.

“In the society that we live in, the men govern the house and if he does not have 1,000 [USh] then the method will not be there.” (Female participant, Shanti)

“That’s why when it comes to financial problems in Uganda, most especially in rural areas, men are the finance controllers, they are the bread winners. So, most women don’t work. So, if you need a service you need to ask for money to facilitate you, even if the service is free. But you need to facilitate. So, to [inaudible] financial problem, men will need to be sensitized about their role in the use of contraceptives. And among that, the financial roles as well because even if the woman is facilitated financially, [if she is also] well educated [about contraception] everything is fine.” (Male participant, RHU)

The participants at RHU explained that some men might believe that taking contraception would enable their wives to have an affair. Stigmas, misconceptions, religious or cultural values all have an impact on men’s perceptions of family planning, and it is not uncommon for wives to take contraception without their husbands knowing.

“They have their reasons, and some of them most especially those in rural areas, they think that when a woman takes family planning, she’ll go find another man out of their marriage, because a woman is not fearing to get pregnant”. (Male participant, RHU)

“Yes. There are times when something is going to be costly, we [men] try to hesitate. Because you have to give transport [money], then we talked about that pregnancy test, and mostly our women sometimes … I don’t know how much it happens on that side but something that is for 3,000, someone can tell the husband no it is at 10,000 and they will increase the cost to the husband maybe because they want to keep something, so they increase themselves. So, at times, when someone looks at it and says ‘you’re telling me its 3,000 and then the transport and then the test, ah don’t go. You stay.” (Male Participant, Shanti)

“But some of them do not know anything about family planning and they don’t want to know. They won’t even approach Shanti’s gate. But the few who want to know, they can support their women.” (Female participant, Shanti)”

“It looks so awkward when you ask a woman to join family planning and she tells you ‘I need confirmation from my husband’, yet it is you the mother carrying the womb. Most of the negative consequences, mothers face. They rarely appear to men, so when we are doing awareness, most especially include men also.” (Female participant, Shanti)

A woman might request to be counselled on family planning at RHU without their husbands’ knowledge about once a week. Issues of finances and payment complicate these clients’ predicament further if they have limited financial resources at their control. For various reasons men in the community may not see family planning as a worthwhile financial investment, leaving enormous opportunity to sensitize men about the positive economic benefits of family planning. One of the participants described the mindset some community members have:

“Okay we feel like, if it is at all free, the men at times will accept it ‘ah okay, it is free, I don’t have anything I can give you so you can go’. When a wife consults him about whether she should come for it or not, he asks what is needed ‘nothing’? Okay you go.” (Male participant, Shanti)

“Of course, what you could put in mind is that although my colleague is talking about African men but … what I have discovered is that African men they do not want to put something in for health, most specially for those contraceptives. If it’s for free they can accept it, but to pay … ah they don’t like that.” (Male participant, Shanti)

The participants believe that emphasizing the financial benefit of contraception may motivate men in the community to consider how family planning could benefit the physical, mental and economic health of their families. One of the participants at RHU reflected on how they used to have door-to-door sensitization outreach efforts.

“And a lot of emphasis has been put on women, training, sensitization, media issues, but we need to come out with the same pace of sensitization of men. I remember some time back at RHU, we used to have door to door sensitization. Move to the workplace, you find two men are digging, two men are working on carpentry, (inaudible), markets, with [RHU] you crack a discussion and by the end of the day, the information is caught. So, then men understand family planning.” (Male participant, RHU)

## Discussion

### Perceived barriers: side effects and the socio-cultural context

For the purpose of this study, social barriers refer to a lack of knowledge and misconceptions, religiosity, cultural values, and stigma. While side effects are potential physiological reactions to hormonal contraceptives and are not exactly borne of the social context like the other barriers mentioned, they have a particular significance in this socio-cultural context, and it is worth exploring how they nuance the other barriers. Side effects were reported to be a significant motivational barrier for the uptake and continuation of contraceptives in each focus group. Side effects, misconceptions, and stigma have a compounding effect on each other; in essence, side effects validate stigma and fuel misconceptions. Side effects such as prolonged bleeding, intrusive procedures, pain, and amenorrhea are inconvenient to the clients, and significantly diminish the perceived value clients place on family planning, especially when side effects legitimize social stigmas and disinformation circulates the community.

When examining some of the misconceptions the community members have such as a fear of permanent infertility, or suspicion of the government’s hidden agenda, it is clear they are reflective of sociocultural values surrounding fertility. Even the clients’ preference of Depo-Provera and the relatively poor reputation of methods like the IUD or the implant demonstrate the socio-cultural perception of contraceptives. Women are forced to navigate complex and interconnecting barriers such as stigma and disinformation surrounding these methods, and while this does not always physically bar them from accessing the clinic, the social stigma against contraception is enough to be a strong demotivating factor and may create risk. Therefore, it is understandable that women feel more comfortable with Depo-Provera because it is as an injection, a procedure they are well familiar with, and it has mild side effects. Most importantly, women can discreetly use the method and access the service from a local VHT in private. Additionally, the intrusive nature of the IUD and the implant are understandably unsettling for the client, especially considering the pain associated with insertion and removal. Considering the other social factors impacting a woman’s decision-making process to access contraception, undergoing a painful IUD insertion procedure would discourage many. Finally, some of the clients are uncomfortable with the idea of some of the long-term side effects, such as amenorrhea. The absence of their period may cause unease for some of the clients, as menstruation is a familiar part of reproduction, and the prospect of losing that natural function of the body is daunting.

### Perceived benefits to modern contraception: urban vs. rural

There are clear and measurable benefits to the uptake of contraception such as lowering maternal and infant mortality, and improved socio-economic status of women. However, there are significant perceived benefits from the urban Ugandan perspective that do not translate into benefits in the rural context. As a participant at RHU explained, urban families who work and earn more money see the economic benefits of family planning and having smaller families, particularly because of the higher cost of living in cities and towns. Yet in the rural areas, often religious and conservative, communities hold stigma against contraception as they see it as interference with God’s will or as a devaluation of fertility. This is critical to highlight as western theories and influences in international development have historically been insensitive to the socio-cultural contexts in which they operate [46]. African Feminist theories emphasize that traditional socio-cultural values are perceived differently among different classes of women, with communities living in poorer rural areas often holding more conservative and traditional values surrounding the family and procreation. More developed urban areas in Uganda see a higher uptake in contraception because the economic benefits of having a smaller family resonate with urban families. Yet to rural communities, the prospect of smaller families, as well as the perceived interference with God’s will translate to increased stigma against modern contraception and the women who use it. The perceived benefits of family planning significantly increase the perceived value of contraceptives for urban Ugandan women, but in rural Uganda the nature of contraception is a quite sensitive and stigmatized topic. This is exemplified when the participants explained that clients were cautious about taking contraception and took care to ensure they used methods that were discreet such as the Depo Provera injection. When it comes to side effects, the higher rate of contraceptive use in the urban areas demonstrates that the decision to manage side effects is an easier trade-off for the prevention of pregnancy for urban Ugandan women. For women in rural areas, inconvenient side effects validate the strong stigma against contraception as well as fuel misconceptions about exaggerated side effects.

### Gender inequality and the importance of gender inclusion

Socio-cultural values have consistently been cited as a barrier to contraceptives in Sub-Saharan Africa [10, 16, 47–49]. This study observes these perceived barriers under an African Feminist lens, which seeks to evaluate what aspects of African society unjustly impact women while conserving a sense of identity with African culture and tradition [26]. While certain cultural values may impact a woman’s motivation to begin family planning, for example the desire for a large family as explained by the participants, they are not barriers in the same way that misconceptions, a lack of knowledge, and stigma can be. If a woman does not wish to take contraception because she wishes to continue to have children, this is not a barrier, but simply a question of personal choice. However, if she is unable to access contraception because her husband does not want her to start family planning, or the male dominated government defunds reproductive healthcare, it is a barrier born out of gender inequality. The participants explained that it was common for clients to report that they could not access contraception because her husband does not want her to use it. They also explained that in their culture, it is typically the men who decide how many children to have in the family as well as control the families’ finances. The African Feminist approach to this issue is to examine how gendered power imbalances negatively impact women, and how levelling these power imbalances may empower women and benefit the community as a whole. Emphasis is placed on the traditional harmonious connection between man and woman and their complimentary roles in life and culture. Based on the data collected, the power imbalance between men and women in the home can create a schism between them, leading to distrust and suspicion either about her motivations to start contraception or the amount of money she needs to start it. A renegotiation of the financial and decision-making power in the household can remedy this and can consolidate their complimentary roles and relationship.

It is critical for village health teams to have the resources to sensitize men just as much as women. Gender inequality and men’s stigma against contraception are significant factors why many of these barriers exist and persist. Gendered power imbalances exist in politics, religion, and the household, and they impact women’s use of contraception in many ways. Men in influential political and religious circles can abuse their power for their own ideological benefit at the detriment of the health of the community. In the household, men benefit from holding the financial decision-making powers in the family when he wants more children than his spouse desires. In fact, the hidden costs to contraception (transportation, insertion fees, lab fees, side effect medication, etc.) can create suspicion in the husband about what his wife is doing with the money since he does not engage in family planning with her. Addressing these unequal power dynamics requires cooperative and inclusive sensitization on how family planning can improve the overall health of the community, as well as a renegotiation of financial decision-making within the household. These same principles apply to increasing the political participation of women and the general support of contraception, as government funding determines the amount and quality of the services, medicines, training, and outreach programs provided. None of the other barriers will be address or solved without political support and funding.

#### Implications for future research and policy

Addressing these issues requires strategic planning and funding, which both Shanti and RHU work very hard to do, however the financial barriers they face create challenges for them in the field. It is clear that as an older and publicly funded organization RHU has more resources at its disposal to deliver family planning in the community, but still struggles to have consistent programming and sensitization programs for men. Shanti, which started as an international non-profit, works to fill gaps in the public services provided in rural areas by relying on international donors to subsidize their contraception and fund their services. This represents a fundamental issue within development and international development in particular. Decades of development work and aid has been poured into African countries like Uganda, yet maternal death, a key development indicator, is still among the highest in the world. A state-led development agenda that sees funding distributed fairly across sectors, as the majority of the population get their health services from governmental health organizations, is essential to addressing the unmet need for contraception in rural Ugandan communities. Yet this is nearly impossible to execute while the state must follow Structural Adjustment Programs (SAPs), aid donated to the government is lost due to corruption, and international development organizations fail to adapt their work and research to be culturally and socially conscientious and reflexive [50–52]. These conditions uphold neo-colonial and imperialistic relations between the Global North and the Global South; therefore, development efforts and economic relations must be decolonized. Future cross-cultural researchers should use a reflexive methodology when gathering and analysing qualitative data, minding their own cultural backgrounds and biases based on their socio-economic location when interpreting qualitative data. Furthermore, cross-cultural qualitative studies must use relevant theoretical frameworks appropriate for the cultural context for the study in order to avoid reproducing imperialistic interpretations of the data. New research paths along this topic could explore the impacts of gender inclusive family planning sensitization efforts, and the ways in which internationally imposed economic policies, such as SAPs, impact Uganda’s public spending. The findings of this study are transferable to many contexts in rural communities across sub-Saharan-African, especially in countries with similar legislations and policies surrounding reproductive healthcare services.

The participants in this study, through their hands-on experience and expertise on the cultural context of Luweero, have many ideas on how to improve access to contraception in their community, such as sensitizing men, distributing information material in the local language, free contraception and side effect treatment, and supporting VHTs. If government funding manages to reach the organizations on the ground, private or public, dedicated reproductive healthcare staff and village health teams will fill key gaps in service delivery, sensitization efforts, and subsidized contraceptives in order to serve Ugandan women with their reproductive autonomy.

## Conclusion

This study sought to explore how various perceived barriers impact the clients and service delivery at Shanti birth house and Reproductive Health Uganda by holding focus group discussions with healthcare workers. African feminisms inform the theoretical framework for this study and addressing gender inequality in rural Uganda, demonstrating the need for gender inclusive sensitization, a renegotiation of the decision-making power within households, and community led state-funded development. We found nuances and connections between key barriers such as the financial burdens, the impact of side effects, socio cultural values and religion, as well as misconceptions and stigma. One of the key underlying reasons for many of these barriers is gender inequality, because there are key actors in the contraception debate with political or social power who benefit from ignoring or exacerbating access barriers to contraception. They are the people of influence in (almost always male-dominated) political and religious circles who do not support family planning for ideological reasons, and individual men who wish to have more children than his spouse desires. This study examined these access barriers through an African Feminist lens, allowing for a more culturally sensitive analysis that found how socio-cultural factors interact with other perceived barriers such as side effects to influence rural Ugandan women’s perceived value of family planning. These findings can help inform current and future outreach educational programs that specifically target men and other community leaders, ideally as part of a state led and state-funded development agenda.

### Strengths and limitations

One of this study’s strengths is that it is consistent with evidence from previous studies on similar topics, such as the impacts of stigma against contraception, misconceptions about contraception, transportation costs to clinics and gender inequality. We had enough data from participants in multiple healthcare occupations and managed to reach saturation. The demonstrated reflexive methodology credits the reliability and cultural sensitivity of the interpretation of the data by detailing researcher perspectives and biases, which is not common practice when western researchers do qualitative research in the African context, though it should be.

A limitation of this study was that we did not collect other pertinent data about the participants’ characteristics that would have provided a wider contextualization of the results, such as occupation, age, and years of experience. Another limitation is that we gained a broad understanding of many different barriers, preventing us from exploring any of the barriers in deeper detail.

## Supplementary Information

**Additional file 1.** Focus group discussion guide.

## Data Availability

The datasets generated and/or analyzed during the current study are not publicly available due analysis being underway for subsequent publications. They are available from the corresponding author on reasonable request.

## Abbreviations

VHTs: Village Health Teams
IUD: Intrauterine device
COREQ: Consolidated criteria for reporting qualitative research
RHU: Reproductive Health Uganda
