# Supplementary material for: Understanding perceived access barriers to contraception through an African feminist lens: a qualitative study in Uganda
Source: BMC Public Health. 2021 Feb 2;21:267. doi: 10.1186/s12889-021-10315-9 (PMC7852360; doi:10.1186/s12889-021-10315-9)
Supplement: Supplementary file 1 — Additional file 1. Focus group discussion guide. [file 12889_2021_10315_MOESM1_ESM.docx]

**Addressing Access Barriers to Contraception in Luweero, Uganda**

*Focus Group Discussion Guide*

1. What types of modern contraception does your clinic dispense?

Probe: How much do they cost?

2. Does your clinic ever run out of stock/supplies of modern contraception?

Probe: How often?

3. How much governmental funding does the clinic receive for these contraception methods in order to make them more affordable for patients?

4. What are some of the reasons that some patients may not use modern contraception?

5. How often do your patients express that the reason they do not use modern contraception is because they cannot afford it?

6. How often are patients able to fully afford the contraception they want?

7. Tell me about a time when a patient was not able to access contraception, how did that affect them?

8. What changes do you believe need to happen in order to help with the shortage of supplies and financial barriers?

9. How would subsidized contraception impact patients?

10. How would subsidized contraception impact the clinic?

11. If you were to see improvements in access to modern contraception at your clinic, what would that look like?

12. What about your current process for dispensing contraception works well?

13. What could be improved on in your current process for dispensing contraception?

14. If you were to make a change to the process, what change would you make? (e.g. better supply, better subsidies, etc.)
